# Risk, Predictors, and Outcomes of Acute Kidney Injury in Patients Admitted to Intensive Care Units in Egypt

**DOI:** 10.1038/s41598-017-17264-7

**Published:** 2017-12-07

**Authors:** Samar Abd ElHafeez, Giovanni Tripepi, Robert Quinn, Yasmine Naga, Sherif Abdelmonem, Mohamed AbdelHady, Ping Liu, Matthew James, Carmine Zoccali, Pietro Ravani

**Affiliations:** 1 Epidemiology Department, High Institute of Public health, Alexandria University, Alexandria, Egypt; 2 Departments of Medicine and Community Health Sciences, University of Calgary, Calgary, Canada; 3 CNR-IFC Clinical Epidemiology and Pathophysiology of Renal Diseases and Hypertension Unit, Ospedali Riuniti, Reggio Calabria, Italy; 4 Internal Medicine Department (Nephrology Unit), Faculty of Medicine, Alexandria University, Alexandria, Egypt; 5 Critical Care Department, Faculty of Medicine, Alexandria University, Alexandria, Egypt; 6 Anesthesia and Surgical ICUs Department, Faculty of Medicine, Alexandria University, Alexandria, Egypt; 7 Departments of Medicine and Community Health Sciences, Libin Cardiovascular Institute of Alberta, O’Brien Institute for Public Health, University of Calgary, Calgary, Canada

## Abstract

Epidemiology of acute kidney injury (AKI) in developing countries is under-studied. We evaluated the risk and prognosis of AKI in patients admitted to intensive care units (ICUs) in Egypt. We recruited consecutive adults admitted to ICUs in Alexandria Teaching Hospitals over six months. We used the KDIGO criteria for AKI. We followed participants until the earliest of ICU discharge, death, day 30 from entry or study end. Of the 532 participants (median age 45 (Interquartile range [IQR]: 30–62) years, 41.7% male, 23.7% diabetics), 39.6% had AKI at ICU admission and 37.4% developed AKI after 24 hours of ICU admission. Previous need of diuretics, sepsis and low education were associated with AKI at ICU admission; APACHE II score independently predicted AKI after ICU admission. A total of 120 (22.6%) patients died during 30-day follow-up. Compared to patients who remained AKI-free, mortality was significantly higher in patients who had AKI at study entry (Hazard Ratio [HR] 2.14; 95% Confidence Interval [CI] 1.02–4.48) or developed AKI in ICU (HR 2.74; 95% CI 1.45–5.17). The risk of AKI is high in critically ill people and predicts poor outcomes. Further studies are needed to estimate the burden of AKI among patients before ICU admission.

## Introduction

Acute Kidney Injury (AKI) affects over 13 million people per year globally, and results in 1.7 million deaths^1,2^. AKI is diagnosed in up to 20% of hospitalized patients^3^ and in 30–60% of critically ill patients^4–7^. It is the most frequent cause of organ dysfunction in intensive care units (ICUs) and the occurrence of even mild AKI is associated with a 50% higher risk of death^8^. AKI results in a significant burden for the society in terms of health resource use during the acute phase, and the potential long-term sequelae including development of chronic kidney disease (CKD) and kidney failure^9–12^.

Four in five cases of AKI occur in the developing world^1,2^. Geographical, etiological, cultural, and economic reasons may underlie potential disparities in the risk of AKI between and within higher and lower income countries. In developing countries, the risk of AKI varies between urban and rural areas, by season and cultural mores, and according to the distribution of infectious agents. The risk and prognosis of AKI vary with the availability of transportation services and health care resources, including medications, equipment, trained personnel, and dialysis facilities^13,14^. The International Society of Nephrology has called the nephrology and the broader health care community to work collaboratively to develop effective programs to stem the tide of preventable deaths due to untreated AKI in developing countries. The “0 by 25” initiative has been launched with a goal that no one should die of untreated AKI by 2025^15^. One major barrier to these initiatives is the limited information about the epidemiology of AKI in developing countries^3,16^. Accurate estimates of the risk of AKI and factors affecting AKI-related outcomes in low-resource regions are key steps towards the design and implementation of initiatives to reduce AKI-related morbidity and mortality.

To address this knowledge gap, we estimated the risk of AKI at the time of admission and after admission to ICU in the four Alexandria Teaching Hospitals. We sought to determine which admission diagnoses were more likely to be associated with AKI and define the risk profile of AKI in ICU. We also studied the prognosis of AKI in this high-risk population.

## Results

### Patient characteristics

**Figure 1:**
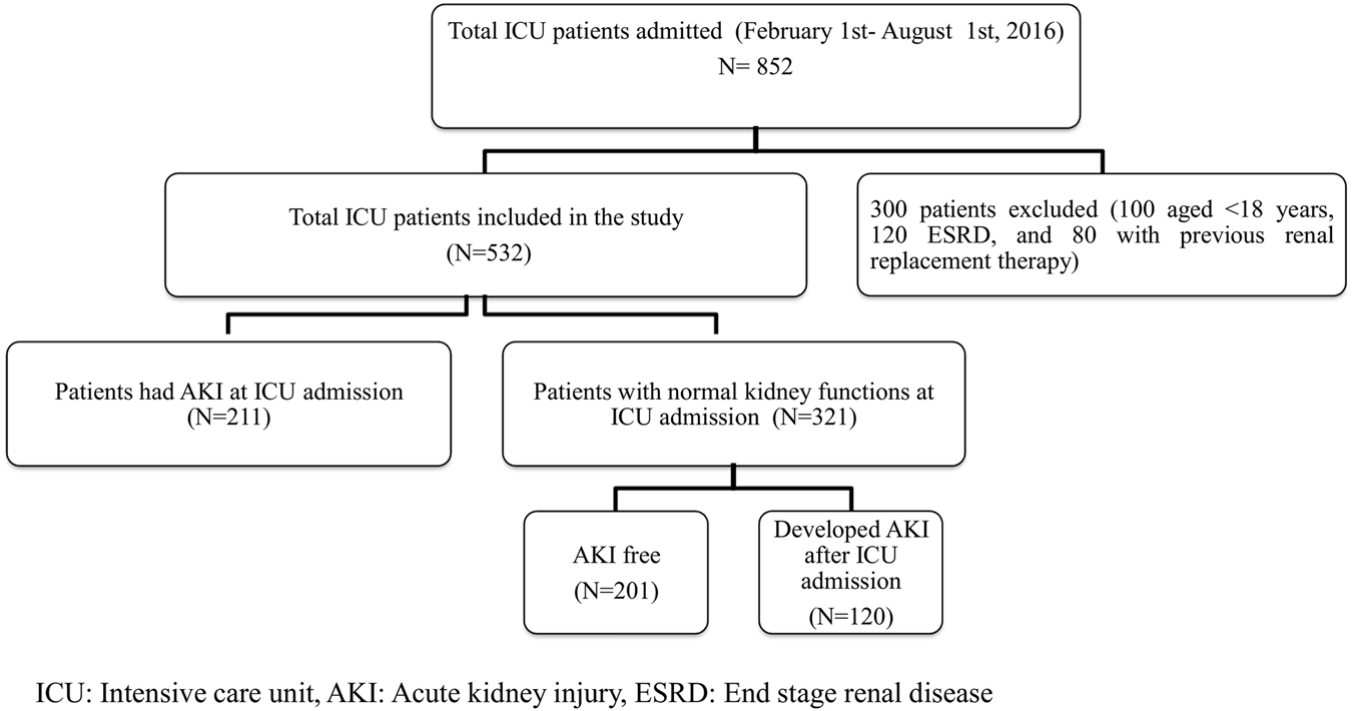
Flow chart of the study cohort.

The study flow-chart is summarized in Figure 1. Table 1 summarizes the baseline characteristics of the study cohort. The median age of the study participants (N = 532) was 45 (Interquartile range [IQR]: 30–62) years, 41.7% were males, 23.5% were smokers, 23.1% were diabetics, 44.2% had cardiovascular diseases and 11.3% had pre-existing CKD. The main ICU admission diagnoses were neurological disorders (14.3%), trauma (14.1%), and pulmonary diseases (14.1%). More than half of the patients were admitted to surgical ICUs (58.3%). Almost two- thirds (62.2%) of the study patients had AKI. Patients with AKI were older, less educated, had a higher burden of co-morbidities, and higher sepsis proportion compared to patients without AKI.

**Table 1:** Baseline characteristics of the study cohort.

**Baseline characteristics**	**Total ICU patients**	**AKI free patients**	**AKI patients (at or after admission to ICU)**	**P value**
**Number of patients**	532 (100)	201 (37.8)	331 (62.2)	
**Age (years)**	45 (30–62)	38 (27–54)	50 (33–65)	<0.001
**Male (%)**	222 (41.7)	74 (36.8)	148 (44.7)	0.07
**Ex-smokers (%) Current smokers (%)**	64 (12) 61 (11.5)	14 (7) 27 (13.4)	50 (15) 34 (10.3)	0.005 0.27
**Marital status** Single (%) Married (%) Divorced (%) widowed (%)	71 (13.3) 436 (82) 4 (0.8) 21 (3.9)	36 (17.9) 159 (79.1) 2 (1) 4 (2)	35 (10.6) 277 (83.7) 2 (0.6) 17 (5.1)	0.03
**Education** Received some forms of education	351 (66)	147 (73.1)	204 (61.6)	0.007
**Residence** Urban Rural	273 (51.3) 259 (48.7)	97 (48.3) 104 (51.7)	176 (53.2) 155 (46.8)	0.27
**Type of ICU unit** Surgical Medical	310 (58.3) 222 (41.7)	146 (72.6) 55 (27.4)	164 (49.5) 167 (50.5)	<0.001
**Co- morbidities** Diabetics Cancer Liver disease CVD Pre-existing CKD COPD	123 (23.1) 65 (12.2) 54 (10.2) 235 (44.2) 60 (11.3) 26 (5)	32 (15.9) 31 (15.4) 11 (5.5) 63 (31.3) 0 10 (4.9)	91 (27.5) 34 (10.3) 43 (13) 172 (52) 60 (18.1) 16 (4.8)	0.002 0.08 0.005 <0.001<0.001 0.94
**History of sepsis at hospital admission Severity of sepsis** Severe sepsis Septic shock	116 (21.8) 60 (51.7) 44 (37.9)	23 (11.4) 16 (69.9) 3 (13)	93 (28.1) 44 (47.3) 41 (44.1)	<0.001
**Reason for ICU admission** Pulmonary GIT CVD Malignancies Infection Neurologic Trauma Obstetric/gynecological disorders Others	75 (14.1) 60 (11.3) 55 (10.3) 54 (10.2) 24 (4.5) 76 (14.3) 75 (14.1) 64 (12) 49 (9.2)	17 (8.5) 24 (11.9) 11 (5.5) 31 (15.4) 2 (1) 29 (14.4) 38 (18.9) 28 (13.9) 21 (10.4)	58 (17.5) 36 (10.9) 44 (13.3) 23 (6.9) 22 (6.6) 47 (14.2) 37 (11.2) 36 (10.9) 28 (8.5)	0.004 0.71 0.004 0.002 0.002 0.94 0.01 0.29 0.44
**Previous use of vasopressors**	111 (20.9)	18 (9)	93 (28.1)	<0.001
**Previous use of diuretics**	74 (13.9)	11 (5.5)	63 (19)	<0.001
**Previous use of Ca channel blockers**	36 (6.8)	6(3)	30 (9.1)	0.007
**Previous use of ACEI**	68 (12.6)	21(10.4)	47 (14.2)	0.21
**Previous use of ARB**	15 (2.8)	5(2.5)	10 (3)	0.72
**Previous use of NSAIDs**	148 (27.8)	55(27.4)	93(28.1)	0.86
**Serum creatinine at ICU admission (mg/dl)**	0.90 (0.66–1.50)	0.70(0.60–0.80)	1.20 (0.80–2.30)	<0.001
**BMI (Kg/m** ^**2**^ **)**	28.79 ± 10.68	28.29 ± 12.97	29.09 ± 9.02	0.40
**Length of ICU stay in days**	6 (3–11)	3 (2–5)	6 (3–9)	<0.001

AKI: Acute kidney injury, CVD: Cardiovascular diseases, CKD: Chronic kidney disease, COPD: Chronic obstructive pulmonary disease, GIT; Gastrointestinal tract, ACEI: Angiotensin converting enzyme inhibitors, ARB: Angiotensin receptor blockade, NSAIDs: Non-steroidal anti-inflammatory drugs, BMI: Body mass index, ICU: Intensive care units.

*Previous use refers to the use at the time of ICU admission.

### AKI at ICU admission

Four in ten people had AKI diagnosed within the first 24 hours of or prior to ICU admission (N = 211; 39.7%). All patients with pre-existing CKD had AKI at study entry. When compared to people without AKI at study entry, people with AKI at ICU admission were older, had a higher burden of co-morbidities, and had a lower level of educational attainment. Pulmonary (20.4%), cardiovascular diseases (14.7%), and obstetric/gynecological disorders (13.3%) were the most common causes for admission to the ICU. Previous use of vasopressors, diuretics, and sepsis were significantly more common in these patients. (Table S1). Half of AKI patients at ICU admission were in stage 2 and the other half were in either stage 1 or 3, and 56.4% required mechanical ventilation during their ICU stay (Table S2).

**Figure 2:**
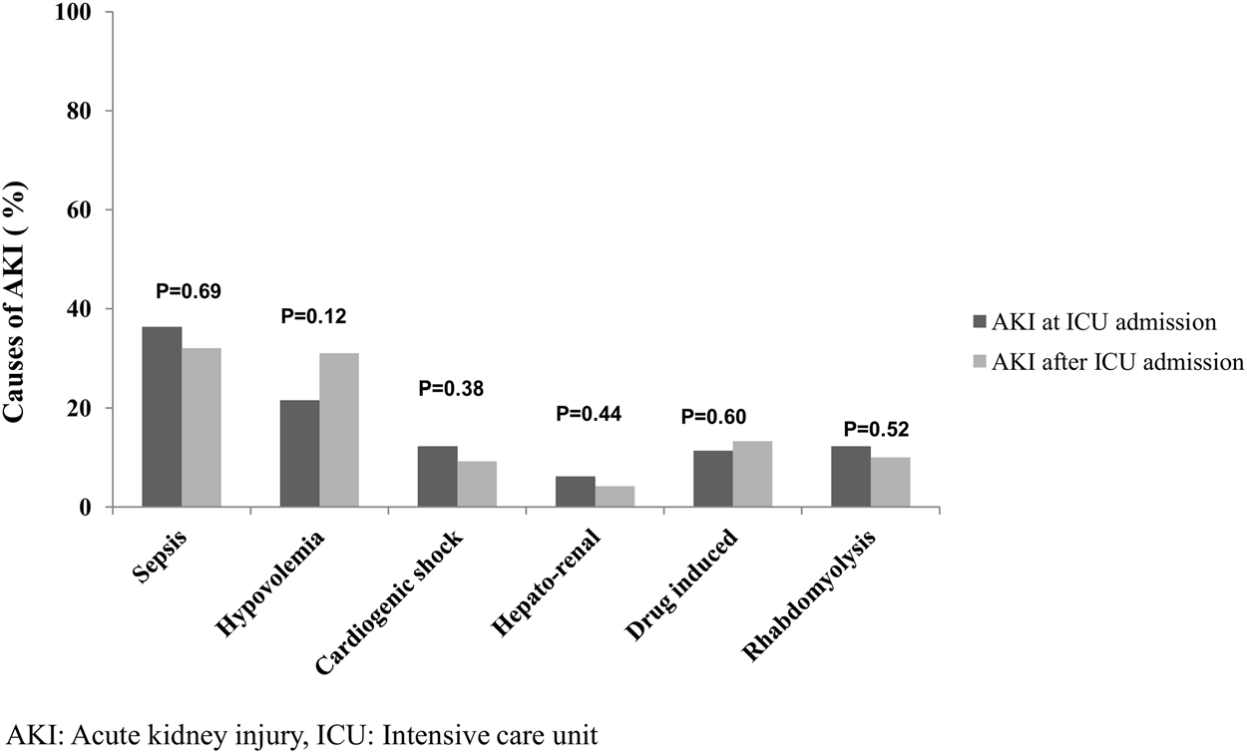
Etiology of AKI by timing of diagnosis: at ICU and after ICU admission.

Sepsis (36%) and hypovolemia (22%) were the most frequent reported etiologies for AKI on ICU admission (Fig. 2). In multivariable analysis, we found the following factors were significantly associated with AKI at study entry: previous use of diuretics (Odds Ratio [OR] 3.17; 95% Confidence Interval [CI] 1.87–5.39), previous use of angiotensin receptor blockers (OR 4.06; 95% CI 1.33–12.38), history of sepsis (OR 2.94; 95% CI 1.87–34.61), and level of education (OR 0.62; 95% CI 0.42–0.92).

### AKI after ICU admission

Of the 321 participants who were AKI-free at study entry, 120 developed AKI after the first 24 hours of ICU admission (37.4%). The most common causes for admission to the ICU were neurological disorders (17.5%) and trauma (15.8%) (Table S3). The majority of patients developed AKI stage 1 (74.2%); 15.8% developed stage 2 and 10% had stage 3. Almost two-thirds (65.5%) of AKI patients required mechanical ventilation during ICU stay (Table S4). Etiologies of AKI after ICU admission were similar to those who had AKI at ICU admission (Fig. 2).

Age, sex, history of cardiovascular disease, obstetric/gynecological cause of admission, type of ICU unit, history of vasopressors and diuretic intake were each associated with AKI (Table S3). In adjusted analysis, only APACHE II score (Hazard Ratio [HR] 1.04; 95% CI 1.03–1.07) significantly predicted the development of AKI after ICU admission.

### Mortality

**Figure 3:**
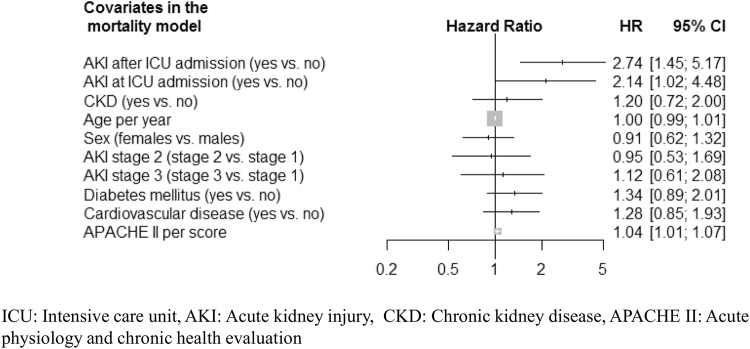
Predictors of 30-day mortality among ICU patients.

Thirty-day mortality was high in this cohort. A total of 120 (22.5%) deaths occurred during 30- days follow-up. Mortality rates were especially high in the first 15 days of ICU stay, with 109 deaths in the first 15 days and 11 deaths in the following 15 days of the study. People with AKI were at increased risk of death, regardless of AKI stage and the timing of AKI occurrence. People with AKI at ICU admission had similar risk of death as those who developed AKI in the ICU (HR 2.74; 95% CI 1.45–5.17 vs. 2.14; 1.02–4.48; P = 0.41). Predictors of mortality were shown in Fig. 3.

### Other outcomes

**Figure 4:**
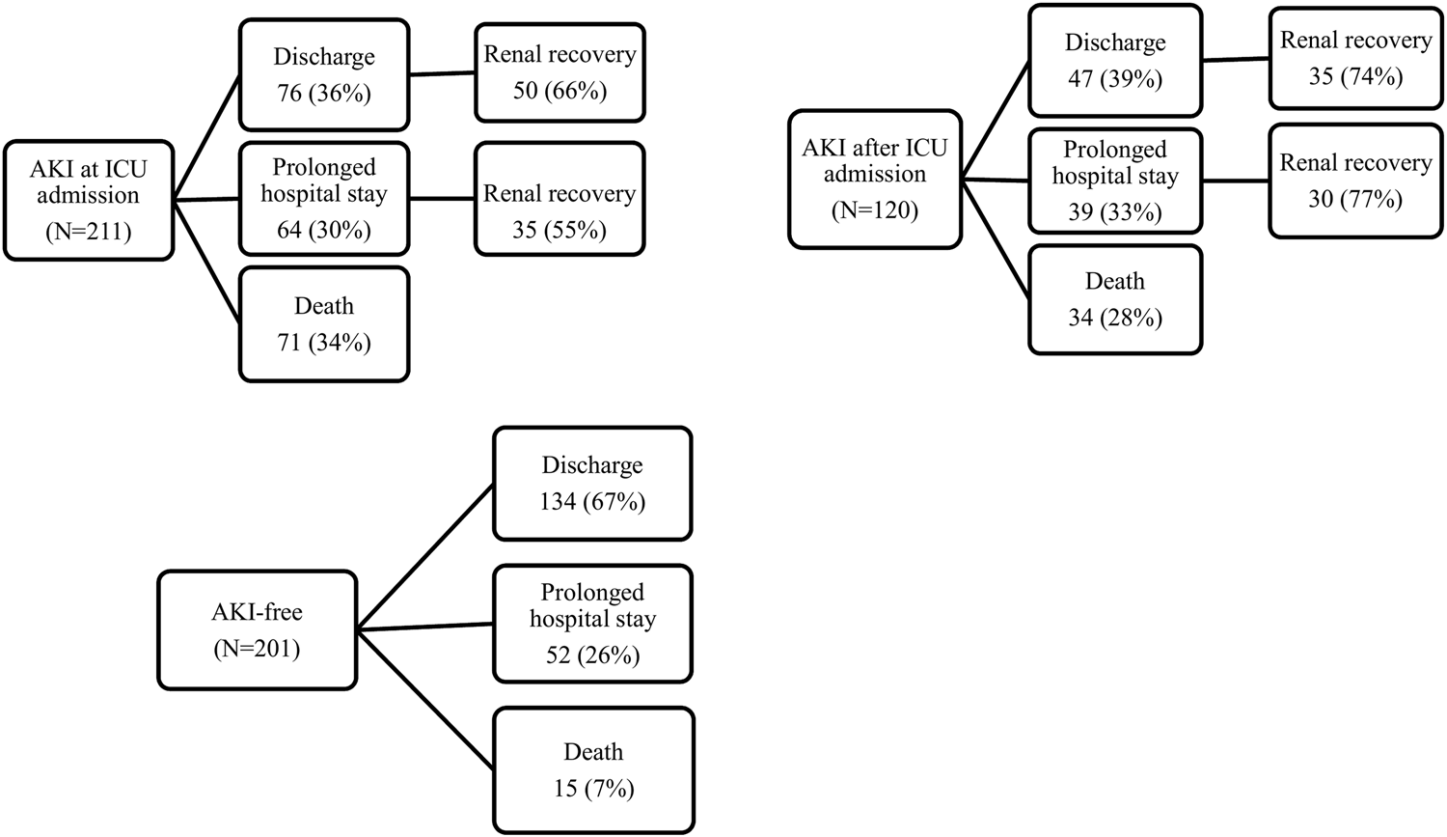
Different outcomes among the study cohort. AKI at ICU admission indicates people diagnosed with acute kidney injury (AKI) when they were admitted to intensive care unit (ICU); AKI after ICU admission indicates people who were AKI free when they were admitted but developed AKI during the study; AKI-free indicates people who remained AKI-free during the study. Hospital stay refers to the need to remain in the hospital beyond 30 days from ICU entry or transfer to another non-ICU unit. Death refers to 30 days mortality. Renal recovery is considered when the last available creatinine on ICU admission fell within 0.3 mg/dl or 50% of the baseline value, without requirements for renal replacement therapy.

A total of 48 patients required renal replacement therapy during ICU stay, 40 (19%) of those with AKI at ICU admission (24 patients with pre-existing CKD and 16 without CKD) and 8 (6%) of patients who developed AKI after ICU admission (P < 0.001). The main causes for renal repalcement therapy were anuria (35%), metabolic acidosis (33%), diuretic-resistant volume overload (19%), and hyperkalemia (13%). About 1 in 3 patients with AKI (vs. 1 in 4 patients without AKI) remained in the hospital beyond day 30 (P = 0.20). A high proportion of AKI patients who remained in hospital for more than 30 days did not fully recover their kidney function at day 30 (1 in 2 people with AKI at ICU admission and 1 in 4 people who developed AKI after ICU admission; P < 0.001). Finally, 31% of the discharged AKI patients did not reach full renal recovery (Fig. 4).

### Sensitivity analysis

When we excluded patients admitted to the ICU during the last month of the study (N = 46) to examine the influence of censoring due to study termination and check consistency of the results, the analyses of predictors for AKI yielded similar results. The outcome of those patients was discharge from ICU (60.9%), death (4.4%), and prolonged hospital stay (19.6%). Only in 7 cases, the study stopped before they reached the study outcome of discharge, death, or prolonged stay. Predictors of mortality remained the same (Table S5).

## Discussion

In this multicenter study of over 500 consecutive patients admitted to ICUs in Alexandria Teaching Hospitals in Egypt, we found that about 40% of patients admitted to ICU had AKI at presentation, and a similar proportion of those who were AKI-free on admission developed AKI during their ICU stay. AKI was associated with high mortality or prolonged hospital stay regardless of its severity and the association between AKI and poor outcomes persisted in analyses that accounted for different potential confounders, including different admission diagnoses and clinical characteristics. Sepsis and hypovolemia were responsible for half of all cases, and in addition to the presence of AKI, only APACHE II score remained an independent predictor of death. These findings suggest that AKI is a common complication in patients admitted to the ICU and portends a poor prognosis.

The risk of AKI among critically ill patients varies widely across studies, usually from 30 to 60%^7,16–18^, although it has been reported to be lower in few studies^19–22^. Variability in the risk of AKI across studies may reflect differences in baseline patient characteristics, criteria used to define AKI (with higher risks reported following the adoption of the new stratification system), study design, and the type of ICUs. The spectrum of severity of AKI may also contribute to data variability^23^. The majority of our AKI patients were in stage 1 or 2, other studies showed higher percentage of AKI patients in more advanced stages, which is associated with outcomes^7,24^. AKI patients included in our study showed clinical characteristics similar to those described from other developing countries^13,25,26^. For example, they were younger, healthier and had higher infection rate than study participants from developed countries^14,27,28^. However, the risk of mortality, impact on length of hospital stay, and the requirement of renal replacement therapy were similar to other studies^29–32^. This finding is consistent with a recent World Health Organization report on lower life expectancy in developing countries, and the potential influence of age in the selection of people that need access to health care^33^.

Consistent with reports from developed countries, our study suggests that AKI in Egyptian ICUs encompasses a complex clinical entity, can complicate different underlying diseases and heralds worse outcomes. Three main findings support this notion. First, different ICU admission diagnoses were associated with AKI in similar ways. Second, in concordance with the multi-factorial pathogenesis of the disease, potential causes of AKI were numerous and similar for AKI diagnosed at or after ICU admission, with sepsis and hypovolemia being the most common. Third, AKI is associated with poor outcomes: one in three ICU patients who experienced AKI died, regardless of whether they had AKI at study entry or developed AKI in ICU. It is possible that AKI contributes to organ damage through the sustained inflammatory response associated with uremia. AKI triggers a cascade of inflammatory processes both locally and systemically^34^. This systemic inflammatory response syndrome is initially characterized by a systemic release of pro-inflammatory cytokines followed by a counter anti-inflammatory response syndrome, which is meant to control the inflammatory process. Uremia in the setting of AKI can disrupt this natural sequence of events potentially contributing to multi-organ failure and subsequently death^35,36^.

The strength of our study lies in the prospective nature of this study, along with the use of the standardized criteria for defining patients with AKI, based on repeated measures of serum creatinine and urine output. Inclusion of all types of ICUs in Alexandria Teaching Hospitals (medical, surgical, obstetric, orthopedics, and cardiac) allows generalization of the results to most ICUs in Egypt. Our study has limitations. First, it was conducted in the teaching hospitals without inclusion of private hospitals or other health sectors. However, given that most critically ill patients are treated in the teaching hospitals, it is unlikely that our findings have underestimated risk and prognosis of AKI. Second, we collected laboratory routine data from all patients admitted to ICUs. While practice may vary across centers, comprehensive data collection increases generalizability. Third, we diagnosed AKI based on nadir serum creatinine due to lack of data on pre-admission renal function. This surrogate for baseline renal function was demonstrated to inflate AKI incidence which in turn leads to misclassification of subsequent death^37^. However, other methods like the admission serum creatinine had the lowest sensitivity for diagnosis of hospital‐acquired AKI^38^ and missed the diagnosis of community‐acquired AKI^39^. For this reason, admission serum creatinine should be used with caution. In only 15% of the patients urine output samples were recorded every 2–6 hours. Fourth, we studied only the short-term effects of AKI and didn’t address its long-term effects. While long-term management of patients with AKI requires a different study design, a short-term follow-up study successfully captures data to address knowledge gaps related to incidence and prognosis of AKI, considering that events tend to occur in the first two weeks of an ICU stay. Finally, this study remains as an observational study with the limiting ability to control for the unmeasured cofounders.

Our study has important implications for health policy and clinical practice. Identifying AKI patients at an early stage is very important to improve prognosis, regardless of AKI severity. Early AKI diagnosis and appropriate treatment are keys to improve outcomes. Several challenges may hinder this prevention strategy in developing countries including Egypt. The major causes of AKI in our cohort, hypovolemia and sepsis, are potentially preventable by early initiation of proper management. However, barriers to early diagnosis and treatment of AKI include limited access to skilled, affordable medical care, cost of antibiotic and fluid resuscitation therapy, availability of renal replacement therapy, and availability of staff and beds in ICUs. Sometimes patients need an ICU bed and there are no available places. Attempts are made to localize an ICU bed in another hospital, but these attempts may fail due to lack of a place or lack of a source for reimbursement. Some patients are transferred, while others keep waiting for a place. All these factors may underlie the high incidence of AKI in our cohort. Raising awareness among the public and health care providers of the importance of prevention, early diagnosis and treatment needs to be followed by policy changes that facilitate investment in resources, infrastructure and facilities. Our data also have implications for future research. Considering that AKI is a determinant of prolonged hospital stay, studies should focus on processes of transfer of care and other hospital outcomes. Finally, long-term follow-up and outcomes of AKI need to be included in future studies, considering the relatively high risk of incomplete recovery of kidney function both in people with prolonged hospital stay and in those who are discharged within 30 days of ICU admission.

In conclusion, the risk of AKI is high among critically ill patients, and health outcomes are poor regardless of the severity of AKI. In people from developing countries who have access to ICUs, the risk and prognosis of AKI are similar to those described in developed countries. Further studies are needed to estimate the burden of AKI among patients before they are admitted to ICUs, their access to ICU services, and long-term prognosis.

## Methods and Materials

### Study design and participants

We conducted a multicenter prospective cohort study in four Alexandria Teaching Hospitals. Alexandria Teaching Hospitals are major teaching hospitals and specialist’s referral centers for the northern part of Egypt. They cover four governorates of Northern Egypt and serve approximately 14 million people. We enrolled all adults who were admitted between February 1^st^ and August 1^st^ 2016 to one of the 10 surgical (5 general surgery and 5 specialized surgical units for neurosurgical, trauma, obstetric/gynecological, cardiothoracic, and orthopedics services) or 8 medical ICUs (3 general medicine and 5 specialized units for burn, renal, cardiac care, neurological, and epilepsy care). The ICUs in the teaching hospitals are all closed model ICUs. We excluded people <18 years of age, those with a history of end-stage kidney failure, and those who had received renal replacement therapy in the preceding 3 months. We followed participants from the ICU admission date until the earliest of discharge, death, transfer to another unit, 30 days from study entry, or study end (August 1^st^, 2016).

### Data collection

We collected information on the dates of hospital admission, and admission and discharge from ICU, demographics, primary ICU admission diagnosis, source of referral, history of co-morbid conditions (diabetes mellitus, hypertension, cardiovascular diseases, malignancies, liver diseases, underlying CKD, and neurological diseases), medication history, and mechanical ventilation during ICU stay. Previous conditions were defined based on medical history, obtained on admission by the treating physician and reviewed by the intensivist on admission to the ICU. Chronic kidney disease was diagnosed clinically following KDIGO guidelines^40^. Sepsis was defined based on medical diagnosis following diagnostic criteria of sepsis reported in International guidelines for management of severe sepsis and septic shock^41^. Hypovolemia was defined by medical history and physical examination of vital signs, skin turgor and central venous pressure measurment^42^. Disease severity on admission to ICU was determined using the acute physiology and chronic health evaluation II (APACHE II) score. Renal replacement therapy was indicated in persistent hyperkalemia, metabolic acidosis not responding to treatment, diuretic- resistant volume overload, and anuria with rising creatinine. Anuria was defined as urine output less than 100 ml in 24 hours. Diuretic resistance was defined as a failure to achieve the therapeutically desired reduction in volume overload despite a full dose of diuretic. Volume overload was assessed clinically using physical examination, central venous pressure measurement and in some cases inferior vena cava diameter measurement by bedside ultrasonography^43^. Contraindications of renal replacement therapy were refractory shock, severe multiple organ failure and severe bleeding tendency. Laboratory data included repeated measures of serum creatinine, urea, electrolytes, liver enzymes and complete blood count.

### AKI and outcomes

#### AKI

AKI was defined according the KDIGO definition, using serum creatinine measurements and urine output criteria (Table S6)^43^. We considered the nadir serum creatinine (creatinine) value obtained within the first seven days of hospital admission as reference creatinine to define AKI, including acute on CKD, because pre-hospital values were not available. Urine output was collected hourly for all the patients, except for 80 patients (15%) for whom collection was every 2–6 hours because of shortage of the staff.

We defined AKI at ICU admission as an AKI diagnosed within the first 24 hours of or prior to study entry (ICU admission). AKI after ICU admission was defined as an AKI that occurred on study day 2 or thereafter (i.e. in people who were AKI-free on study day 1).

#### Outcomes

The outcome of interest was 30-day mortality from all causes. Other outcomes included the need for dialysis in ICU and recovery of kidney function following AKI diagnosis. Participants met the criteria for renal recovery if the last available creatinine on ICU admission fell within 0.3 mg/dl or 50% of the baseline value, without requirement of renal replacement therapies^44^. Prolonged hospital stay was defined as the need to remain in hospital beyond day 30 from ICU entry or transfer to another non-ICU unit.

### Statistical analysis

#### Descriptive statistics

we used mean and standard deviation (SD), medians and inter-quartile ranges or frequencies and percentage, as appropriate. We studied the risk of AKI and its predictors, treating AKI as an outcome; we then studied the prognosis of AKI, treating AKI as an exposure.

#### Risk factors of AKI

We used logistic regression to model the presence of AKI at ICU admission as a function of all covariates defined in the data collection section. We used Cox regression to model time to AKI in people who were AKI-free when they were admitted to ICU.

#### Mortality

We used survival analyses to study time to death, with the date of study entry as time zero, and a time-varying (time-updated) covariate approach for AKI acquired in ICU. When AKI occurred during the study, all time prior to AKI was considered AKI-free; all time after was considered exposed to AKI. For people who already had AKI when they entered the study, all time was considered exposed to AKI from baseline.

#### Model building and checking

We adjusted all models for the variables described in the data collection section. During model building, we checked that results were consistent across categories of age and sex (including formal testing for interaction). We used graphical tests and formal tests based on residual analyses to assess the validity of each model, including the proportional hazard requirement for Cox regression. We used likelihood ratio tests to compare nested models, information criteria for non-nested models, discrimination and calibration for logistic models, and Cox-Snell residuals to graphically assess the goodness-of-fit of survival models. In sensitivity analyses, we excluded participants enrolled in the last month of the study to examine the influence of censoring due to study termination and check consistency of the results. We used STATA (www.stata.com) and SPSS (www.spss.com) for all analyses.

The Institutional Review Board of the High Institute of Public Health and the Faculty of Medicine, Alexandria University, approved the study. All methods were performed in accordance with the relevant guidelines and regulations. Informed written consent was obtained from each study participant or their caregiver as required.

All data generated or analysed during this study are included in this published article (and its Supplementary Information files).

## Electronic supplementary material

Supplementary data
