# Supplementary material for: Risk, Predictors, and Outcomes of Acute Kidney Injury in Patients Admitted to Intensive Care Units in Egypt
Source: Sci Rep. 2017 Dec 7;7:17163. doi: 10.1038/s41598-017-17264-7 (PMC5719418; doi:10.1038/s41598-017-17264-7)
Supplement: Supplementary file 1 — Supplementary data [file 41598_2017_17264_MOESM1_ESM.pdf]

RISK, PREDICTORS, AND OUTCOMES OF ACUTE KIDNEY INJURY IN PATIENTS ADMITTED TO INTENSIVE CARE UNITS IN EGYPT

Samar Abd ElHafeez DrPH*[1, 2]; Giovanni Tripepi* PhD[3]; Robert Quinn MD, PhD, FRCPC[2]; Yasmine Naga MD, PhD[4]; Sherif Abdelmonem MD, PhD[5]; Mohamed AbdelHady MD, PhD[6]; Ping Liu PhD[2]; Matthew James MD, PhD[7]; Carmine Zoccali FASN, FNKF, FERA[3]; Pietro Ravani* MD, PhD, FERA[2]

[1] Epidemiology Department, High Institute of Public health, Alexandria University, Alexandria, Egypt.
[2] Departments of Medicine and Community Health Sciences, University of Calgary, Calgary, Canada.
[3] CNR-IFC Clinical Epidemiology and Pathophysiology of Renal Diseases and Hypertension Unit, Ospedali Riuniti, Reggio Calabria, Italy.
[4] Internal Medicine Department (Nephrology Unit), Faculty of Medicine, Alexandria University, Alexandria, Egypt.
[5] Critical Care Department, Faculty of Medicine, Alexandria University, Alexandria, Egypt.
[6] Anesthesia and Surgical ICUs Department, Faculty of Medicine, Alexandria University, Alexandria, Egypt.
[7] Departments of Medicine and Community Health Sciences, Libin Cardiovascular Institute of Alberta, O'Brien Institute for Public Health, University of Calgary, Calgary, Canada.

#GT and PR co-supervised SA (post-doctoral fellow)

Correspondence:
Name: Dr. Samar Abd ElHafeez
Address: High Institute of Public Health, Alexandria University, 165 ElHorreya Avenue, ElHadara, Alexandria, Egypt
Telephone: +201005052416
Email: samarabdelhafeez.epid@gmail.com
Word count: 3362
Abstract: 199

Running title: AKI in ICUs in Egypt

Table S1: Baseline characteristics of AKI patients at ICU admission

Baseline characteristics	No AKI at ICU admission	AKI at ICU admission	P value
**Number of patients**	321 (60.3)	211 (39.7)	
**Age (years)**	40 (28-75)	53 (32-67)	<0.001
**Male (%)**	134 (41.7)	88 (41.7)	0.99
**Ex-smokers (%)**	36 (11.2)	28 (13.3)	0.48
**Current smokers (%)**	39 (12.1)	22 (10.4)	0.54
**Marital status**			
Single (%)	54 (16.8)	17 (8.1)	
Married (%)	256 (79.8)	180 (85.3)	0.10
Divorced (%)	4 (1.2)	0	0.10
Widowed (%)	7 (1.2)	14 (6.6)	0.01
**Education**			
Received some forms of education	232 (72.3)	118 (55.9)	<0.001
**Residence**			
Urban	163 (50.8)	110 (52.1)	0.95
Rural	158 (49.2)	101(47.9)	
**Type of ICU unit**			
Surgical	217 (67.6)	93 (44.1)	<0.001
Medical	104 (32.4)	118 (55.9)	
**Co- morbidities**			
Diabetics	58 (18.1)	65 (30.8)	<0.001
Cancer	48 (15)	17 (8.1)	0.02
Liver disease	24 (7.5)	30 (14.2)	0.01
CVD	116 (36.1)	119 (56.4)	<0.001
Pre-existing CKD	0	60 (28.4)	<0.001
COPD	19 (3)	7 (3)	0.17
**History of sepsis at hospital admission**	44 (13.7)	72 (34.1)	<0.001
**Severity of sepsis**			
Severe sepsis	29(65.9)	31(43.1)	
Septic shock	9(20.5)	35(48.6)	
**Reason for ICU admission**			
Pulmonary	32 (10)	43 (20.4)	0.001
GIT	36 (11.2)	24 (11.2)	0.96
CVD	24 (7.5)	31 (14.7)	0.009
Malignancies	49 (15.3)	5 (2.4)	<0.001
Infection	4 (1.2)	20 (9.5)	<0.001
Neurologic	50 (15.6)	26 (12.3)	0.29
Trauma	57 (17.8)	18 (8.5)	0.003
Obstetric/gynecological disorders	36 (11.2)	28 (13.3)	0.50
Others	33 (10.3)	16 (7.6)	0.29
**Previous use of vasopressors**	40 (12.5)	71 (33.6)	<0.001
**Previous use of diuretics**	27 (8.4)	47 (22.3)	<0.001

**Previous use of Ca channel blockers**	14 (4.4)	22 (10.4)	0.008
**Previous use of ACEI**	38 (11.4)	30 (14.8)	0.42
**Previous use of ARB**	5 (1.6)	10 (4.7)	0.03
**Previous use of NSAIDs**	97 (30.2)	51 (24.2)	0.13
**BMI (Kg/m^2)**	28.42±11.16	29.33±9.90	0.88
**Length of ICU stay in days**	7 (3-12)	6 (3-11)	0.51

AKI: Acute kidney injury, CVD: Cardiovascular diseases, CKD: Chronic kidney disease, COPD: Chronic obstructive pulmonary disease, GIT; Gastrointestinal tract, ACEI: Angiotensin converting enzyme inhibitors, ARB: Angiotensin receptor blockade, NSAIDs: Non-steroidal anti-inflammatory drugs, BMI: Body mass index, ICU: Intensive care units

*Previous use refers to the use at the time of ICU admission

Table S2: Clinical characteristics of AKI at ICU admission (study entry; N=211)

Clinical characteristics	Frequencies
**AKI stages**	
Stage 1	47 (22.2)
Stage 2	105 (49.8)
Stage 3	59 (28)
**Mechanical ventilation**	119 (56.4%)
**FiO2 (%)**	40 (21-60)
**PaO2**	80 (67-98)
**Vaosactive therapy**	71 (33.6)
**Glasgow coma score**	12 (8-15)
**APACHE II**	20.67±8.46
**Platelets (x10^3/µl)**	188 (130-257)
**Urine output(ml/kg/hr)**	0.49 (0.28-1.05)
**ALT (U/L)**	29 (19-84)
**AST (U/L)**	40 (23-84)
**Serum creatinine at ICU admission (mg/dl)**	1.84 (1.25-3.80)
**Serum creatinine at ICU discharge (mg/dl)**	0.82 (0.65-1.20)
**Na(mmol/l)**	137±7.72
**K(mmol/l)**	4.1±0.85
**Blood urea (mmol/l)**	4.34 (2.67-7.18)

AKI: Acute kidney injury, ALT: Alanine aminotransferase, AST: Aspartate aminotransferase, APACHE II: Acute Physiology and Chronic Health Evaluation

*Use of mechanical ventiation during the whole ICU stay

Table S3: Baseline characteristics for AKI after ICU admission

Baseline characteristics	AKI free	AKI after ICU admission	P value
**Number of patients**	201 (62.6)	120 (37.4)	
**Age (years)**	38 (27-54)	46 (34-62)	0.01
**Male (%)**	74 (36.8)	60 (50)	0.02
**Ex-smokers (%)**	14 (7)	22 (18.3)	0.002
**Current smokers (%)**	27 (13.4)	12 (10.1)	0.36
**Marital status**			
Single (%)	36 (17.8)	18 (15.1)	
Married (%)	159 (79.1)	97 (80.8)	0.71
Divorced (%)	2 (1)	2 (1.7)	0.60
Widowed (%)	4 (2)	3 (2.5)	0.76
**Education**			
Received some forms of education	147 (73.1)	85 (70.6)	0.66
**Residence**			
Urban	97 (48.3)	66 (55)	0.24
Rural	104 (51.7)	54 (45)	
**Type of ICU unit**			
Surgical	146 (72.6)	71 (59.2)	0.01
Medical	55 (27.4)	50 (40.8)	
**Co- morbidities**			
Diabetics	32 (15.9)	26 (21.7)	0.20
Cancer	31 (15.4)	17 (14.2)	0.76
Liver disease	11 (5.5)	13 (10.8)	0.08
CVD	63 (31.3)	53 (44.2)	0.02
COPD	10 (5)	9 (8)	0.35
**History of sepsis at hospital admission**	23 (11.4)	21 (17.5)	0.13
**Severity of sepsis**			
Severe sepsis	16 (69.6)	13 (61.9)	
Septic shock	3 (13)	6 (28.6)	
**Reason for ICU admission**			
Pulmonary	17 (8.5)	15 (12.5)	0.24
GIT	24 (11.9)	12 (10.0)	0.59
CVD	11 (5.5)	13 (10.8)	0.08
Malignancies	31 (15.4)	18 (15.0)	0.92
Infection	2 (1)	2 (1.7)	0.60
Neurologic	29 (14.4)	21 (17.5)	0.46
Trauma	38 (18.9)	19 (15.8)	0.49
Obstetric/ gynecological disorders	28 (13.9)	8 (6.7)	0.04
Others	21 (10.4)	12 (10.1)	0.90
**Previous use of vasopressors**	18 (9)	22 (18.3)	0.01
**Previous use of diuretics**	11 (5.5)	16 (13.3)	0.01
**Previous use of Ca channel**	6 (3)	8 (6.7)	0.12

**blockers**			
**Previous use of ACEI**	21 (10.4)	17 (14.2)	0.32
**Previous use of ARB**	5 (2.5)	0 (0)	0.08
**Previous use of NSAIDs**	55 (27.4)	42 (35)	0.15
**BMI (Kg/m^2)**	28.29±12.70	28.66±7.21	0.78
**APACHE II**	12.23±5.66	15.46±5.54	<0.001
**Length of ICU stay in days**	5 (2-10)	9 (5-15)	<0.001

CVD: Cardiovascular diseases, CKD: Chronic kidney disease, COPD: Chronic obstructive pulmonary disease, ACEI: Angiotensin converting enzyme inhibitors, ARB: Angiotensin receptor blockade, NSAIDs: Non-steroidal anti-inflammatory drugs, BMI: Body mass index, APACHE II: Acute Physiology and Chronic Health Evaluation

*Previous use refers to the use at the time of ICU admission

Table S4: Clinical characteristics of AKI after ICU admission (N=120)

Clinical characteristics	Frequencies
**AKI stages**	
Stage 1	89 (74.2)
Stage 2	19 (15.8)
Stage 3	12 (10)
**Mechanical ventilation**	78 (65.5)
**FiO2 (%)**	40 (38-53)
**PaO2**	93 (82-93)
**Vaosactive therapy**	44 (30.6)
**Glasgow coma score**	11 (9-15)
**APACHE II**	15.39±5.21
**Platelets (x10^3/µl)**	208 (148-287)
**Urine output (ml/kg/hr)**	0.49 (0.68-1.33)
**ALT (U/L)**	32 (21-60)
**AST (U/L)**	41 (33-60)
**Serum creatinine at ICU admission (mg/dl)**	0.80 (0.65-1.00)
**Serum creatinine at ICU discharge (mg/dl)**	0.82 (0.65-1.20)
**Na (mmol/l)**	137±7.72
**K (mmol/l)**	4.1±0.85
**Blood urea (mmol/l)**	4.34 (2.67-7.18)

ALT: Alanine aminotransferase, AST: Aspartate aminotransferase, APACHE II: Acute Physiology and Chronic Health Evaluation

*Use of mechanical ventiation during the whole ICU stay

Table S5: Predictors of mortality in sensitivity analysis

Variables	Hazard ratio	95% confidence interval
AKI at ICU admission	2.29	(1.09-4.82)
AKI after ICU admission	2.89	(1.53-5.48)
Age (years)	1.00	(0.98-1.01)
Sex	0.91	(0.62-1.33)
History of CKD	1.20	(0.71-2.004)
History of diabetes mellitus	1.45	(0.96-2.19)
History of cardiovascular disease	1.22	(0.81-1.86)
AKI stage 2$	0.87	(0.49-1.56)
AKI stage 3$	0.99	(0.80-1.21)
APACHE II	1.04	(1.01-1.07)

AKI: Acute kidney injury, ICU: Intensive care unit, CKD: Chronic kidney disease, APACHE II: Acute Physiology and Chronic Health Evaluation

$ AKI stage 1 is the reference group

*In the model of mortality, both AKI and APACHE II maintained similar association with the outcome

Table S6: KDIGO definition and staging of AKI

**AKI is diagnosed in the presence of:**<br>• Increase in serum creatinine by ≥ 0.3 mg/dl within 48 hours; or<br>• Increase in serum creatinine to ≥ 1.5 times baseline, which is known or presumed to have occurred within the prior 7 days; or<br>• Urine volume < 0.5 ml/kg/h for 6 hours.		
**Stage**	**Serum creatinine**	**Urine output**
1	1.5–1.9 times baseline, or ≥ 0.3 mg/dl increase	< 0.5 ml/kg/h for 6–12 hours
2	2.0–2.9 times baseline	< 0.5 ml/kg/h for ≥ 12 hours
3	3.0 times baseline, or increase in creatinine to ≥ 4.0 mg/dl, or initiation of renal replacement therapy	< 0.3 ml/kg/h for ≥ 24 hours OR anuria for ≥ 12 hours

KDIGO: Kidney Disease Improving Global Outcome[43]
